# Robotic versus traditional laparoendoscopic single-site radical hysterectomy with no-manipulation technique: a retrospective cohort study

**DOI:** 10.3389/fonc.2026.1643025

**Published:** 2026-02-17

**Authors:** Yu Chen, Lusi Deng, Jianhong Liu, Fan Yang, Qiao Wang, Sijing Chen, Xu Yang, Ying Zheng

**Affiliations:** 1 Department of Gynecologic Oncology, West China Second University Hospital, Sichuan University, Chengdu, China; 2 Key Laboratory of Birth Defects and Related Diseases of Women and Children (Sichuan University), Ministry of Education, Chengdu, China

**Keywords:** cervical cancer, laparoendoscopic single-site surgery, no-manipulation technique, radical hysterectomy, robotic single-site surgery

## Abstract

**Background and objectives:**

Controversies and challenges over minimally invasive approach in performing radical hysterectomy for early-stage cervical cancer have been raised. This study aimed to determine whether robotic approach was superior to traditional single-site radical hysterectomy with no-manipulation technique for early-stage cervical cancer.

**Methods:**

Patients diagnosed with FIGO 2018 stage IB1, IB2 or IIA1 cervical cancer who underwent robotic or traditional single-site radical hysterectomy and pelvic lymphadenectomy between April 2019 and December 2023 were enrolled to assess the perioperative and survival outcomes.

**Results:**

73 patients were included in the robotic group and 51 cases in the traditional group. The robotic group had significantly shorter operative time (236.5 ± 52.8 *vs* 390.8 ± 73.5 min, p<0.001), less blood loss (50.0 *vs* 150.0 mL, p<0.001), and shorter drainage time (3.7 ± 1.2 *vs* 4.7 ± 1.9 days, p=0.001), with a tendency of less conversions and complications compared to the traditional group. The two groups exhibited comparable 3-year disease-free survival (89.8% *vs* 95.8%, p=0.399) and overall survival (95.8% *vs* 96.3%, p=0.752) rates.

**Conclusion:**

Robotic and traditional single-site radical hysterectomies with no-manipulation techniques are both feasible and safe for early-stage cervical cancer with comparable survival outcomes, though longer follow-up is needed to confirm non-inferiority. The robotic system could significantly reduce surgical difficulties and improve perioperative outcomes.

## 1 Introduction

Cervical cancer ranks fourth in the incidence of cancer with an estimated 661,021 new cases emerging in 2022, and is also the fourth leading cause of cancer-related mortality among women globally (1). Radical hysterectomy and pelvic lymph node dissection (PLND) is recommended as the standard treatment for patients with stage IB1, IB2 and IIA1 using the International Federation of Gynecology and Obstetrics (FIGO) staging system (Version 2018) (2). Over the past decades, minimally invasive surgery (MIS) has been widely adopted, offering benefits such as reduced blood loss, shorter hospital stays, and faster recovery compared to open laparotomy.

The publication of the Laparoscopic Approach to Cervical Cancer (LACC) trial in 2018, which reported inferior survival outcomes for MIS compared to laparotomy, raised controversies and technical refinement within this field (3). The results might ascribe to squeezing and damage to the cervical lesion by uterine manipulator, intraperitoneal CO2 circulation, and unsealed colpotomy leading to tumor cell growth and dissemination. In response, researchers have tried to find improvements against these factors rather than directly abandon it. Studies have proved that the survival outcomes of patients with cervical lesions smaller than 2 cm after MIS were not inferior to those undergoing laparotomy (4, 5). In addition, researchers also explored technical innovations like no-manipulation techniques or gasless laparoscopy, and strictly followed the tumor-free principle including sealed colpotomy, contained tissue extraction, and repeated pelvic irrigation to take full advantages of MIS without compromising oncological outcomes (6–8). Moreover, surgeons’ experience and skills determine the excision adequacy of radical hysterectomy, which is also one of the most important prognostic factors.

Laparoendoscopic single-site surgery (LESS), which enters into the peritoneal cavity through a single umbilical incision or vagina, has been greatly appreciated in gynecology because it shows advantages in minor invasion, quick and safe specimen extraction, alleviated pain, fast recovery, as well as satisfying patients’ expectation in cosmesis (9). However, the omission of a uterine manipulator introduces significant technical challenges, particularly in achieving adequate exposure for precise parametrial dissection and systematic lymphadenectomy. These challenges are markedly exacerbated in the LESS approach due to its inherent ergonomic constraints, such as loss of triangulation, instrumental collision, and a limited ability for surgical assistance.

The emerging robotic surgical system, characterized by magnified three-dimensional (3D) camera, articulated instruments, and ergonomic surgeon console, presents a potential solution to these amplified technical difficulties. These features enhance visual clarity, operative dexterity, precision and reduce surgeons’ fatigue to facilitate complex dissection in the confined single-site workspace, making the complementation of a no-manipulation radical hysterectomy more feasible.

While several studies have described the feasibility of both traditional LESS (10) and robotic laparoendoscopic single-site surgery (R-LESS) in managing early-stage cervical cancer (11–13), a direct comparison between these two approaches with no-manipulation technique is lacking. Therefore, the primary objective of this study was to determine whether R-LESS radical hysterectomy with no-manipulation technique was superior to traditional LESS in the perioperative and survival outcomes for early-stage cervical cancer.

## 2 Materials and methods

### 2.1 Study design and participants

This was a single-center retrospective cohort study that had obtained ethical approval from the Institutional Review Board (IRB number: 2023331). Patients were consecutively enrolled if they were diagnosed with cervical cancer after cervical biopsy or conization, had a clinical FIGO 2018 stage of IB1, IB2, IIA1, and underwent type C (Querleu-Morrow, Q-M) hysterectomy with no-manipulation technique and PLND through transumbilical single-site approach with or without robotic assistance by the same surgeon from April 2019 to December 2023. Patients with other synchronous malignancies were excluded.

Enhanced abdomino-pelvic CT or MRI was conducted before surgery to assess tumor size, parametrial infiltration and lymph node involvement. Sufficient consultations were provided to each patient detailing the advantages and disadvantages of the two approaches, as well as the potential risk in poor survival associated with MIS in radical hysterectomy prior to their final decisions. All patients had provided written informed consent before surgery.

### 2.2 Surgeons and procedures

Procedures were performed by the same surgeon who had extensive experience in LESS since 2018. The da Vinci Xi surgical system (Intuitive Surgical, CA, USA) was introduced to our hospital in May 2021. Under general anesthesia, patients were placed in the Trendelenburg position. A 2-cm central, vertical umbilical incision was made for LESS using an open Hasson technique, while a 3-cm incision was required for R-LESS to accommodate three robotic cannulas and one conventional instrument. After inserting a multichannel port (Kangji Medical, Hangzhou, China) through the umbilical incision, the pneumoperitoneum was established at a pressure of 12–14 mmHg.

For R-LESS preparation, the patient cart was driven to the right side of the operating table. An 8-mm 30-degree robotic camera was docked in Arm 2 towards the pelvic cavity following a comprehensive inspection. Then another two robotic instruments, normally monopolar scissors and fenestrated bipolar forceps, were docked in Arm 1 and 3. Occasionally, other robotic instruments like Maryland forceps or Vessel Sealer were used according to the surgeon’s preferences.

**Figure 1:**
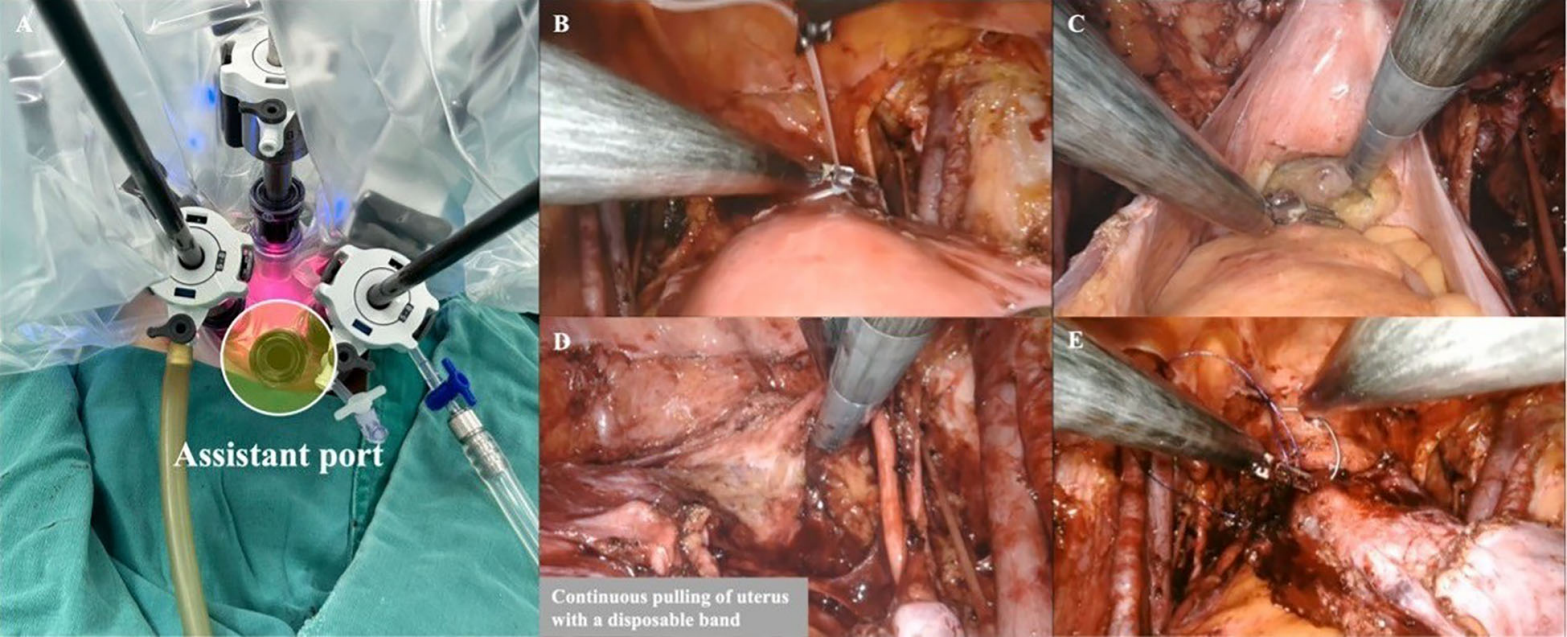
The banding method for R-LESS radical hysterectomy **(A)** Port arrangement; **(B–D)** The banding method for uterine manipulation; **(E)** Seal the vagina before colpotomy.

Patients with tumor size smaller than 2 cm without lymph-vascular space invasion (LVSI) underwent sentinel lymph node dissection (SLND) with indocyanine mapping, and others underwent systematic PLND. Radical hysterectomy was sequentially performed following lymphadenectomy. For conventional LESS, we innovatively developed Zheng’s 4C suspension method using KS needles and sutures to replace the traditional uterine manipulator. This technique involves transabdominal suspension of pelvic tissues such as the vascular peritoneum and ligaments, thereby achieving adequate exposure of the parametrial and lymph node dissecting areas. The detailed procedural steps and efficacy of this method have been thoroughly described and validated in previous publications (Chen et al., 2020; 8, 14). At the initial stage of R-LESS, we continued to employ this suspension method. However, due to its technical complexity and the obstruction caused by robotic arms positioned directly above the abdominal wall, which made adjustments of the suspension sutures considerably more challenging, we subsequently adopted the simplified banding method especially suitable for R-LESS in the following cases. In this technique, a disposable sterile cerclage band is placed around the cervico-isthmic junction and is continuously lifted and laterally retracted by the assistant using laparoscopic forceps through the assistant port. The advantages of this banding method include its simplicity, time-saving nature, elimination of the need for repeated suture adjustments, and the ability to perform precise traction under the magnified view of the robotic camera (**Figure 1**). After ensuring adequate resection margins (parametrium ≥3 ;cm, vagina ≥2 ;cm), a barbed suture was used to seal the vagina before colpotomy for preventing tumor spillage. Then the resected uterus and bagged lymph nodes were directly retrieved through vagina followed by vaginal stump closure in a two-layer continuous manner. Finally, the umbilical incision was plastically repaired using Zheng’s anchor suturing technique (15). Both methods avoid compression of the cervical lesion, adhere to the tumor-free principle, and provide sufficient surgical exposure.

All operations were assisted by a team familiar with both LESS and R-LESS protocols. The assistant’s responsibilities differ between the two approaches. In traditional LESS, the bed-side assistant primarily managed the endoscope, aiming to provide a clear and steady surgical view. In R-LESS, however, the robotic camera was manipulated by the main surgeon directly from the Console, leaving the assistant available to use an additional laparoscopic instrument to aid in exposure, retraction, or hemostasis for simplifying procedures and securing important anatomies. The assistant’s tasks were pre-defined and systematically standardized according to the surgical approach and were consistently applied in every case to minimize variability arising from differences in assistant involvement.

A procedure was considered successfully completed if the entire operation was performed via the planned transumbilical single−site approach without conversion to multiport laparoscopy or laparotomy. Intraoperative complications that were managed intraoperatively without altering the surgical approach were still classified as technical successes.

### 2.3 Collected parameters

Patients’ information was retrospectively collected. Baseline characteristics included age, BMI, menopausal status, gravity and parity, history of abdominal surgery, comorbidity and clinical stage. Surgical parameters included type of no-manipulation technique, extent of lymphadenectomy, operative time (OT), estimated blood loss (EBL), intraoperative complication, conversion, exhaust time, drainage time, indwelling catheter time, pain assessment using Visual Analog Scale (VAS), postoperative length of stay and postoperative complication. Histopathological type, differentiated degree, depth of stromal infiltration, length of resected vagina, LVSI, parametrial involvement, vaginal fornix involvement, positive margin, number of resected nodes, and lymph node metastasis were retrieved from pathological reports, confirming patients’ postoperative disease status. Follow-ups were conducted by outpatient service or telephone every three months until September 2024 for survival analysis.

### 2.4 Statistical analysis

Continuous variables were reported as mean ± standard deviation (SD) or median (P25, P75). Categorical variables were delineated by a precise number with its percentage. For group comparisons, the Student t-test, Mann–Whitney test, Fisher’s exact test, or Chi-square test was selected based on the data distribution and characteristics. To identify independent risk factors associated with disease recurrence and death, univariate and multivariate analyses were performed using Cox proportional hazards regression models. Statistical significance was set at a P value less than 0.05. Data analysis was conducted using SPSS statistics, version 26.0 (IBM, Armonk, NY, USA). A *post hoc* power analysis was performed using G*Power software version 3.1 to assess the adequacy of the sample size for detecting clinically relevant differences in key outcomes.

## 3 Results

A total of 124 patients were included in this study, comprising 73 cases in the R-LESS group and 51 cases in the LESS group. After the introduction of da Vinci Xi system, radical hysterectomies for cervical cancer were mostly performed by R-LESS.

Baseline characteristics were summarized in **Table 1** which were all balanced with no statistical significance between the two groups. The clinical stage distributions of patients were comparable between the two groups (p=0.479). Regarding no-manipulation techniques adopted in the R-LESS group, 17 of 73 cases (23.3%) were performed with Zheng’s 4C suspension method, and the remaining 56 cases (76.7%) utilized the banding method. The width of resected parametrium and length of vagina reached at least 3 cm. As for the extent of lymphadenectomy in the R-LESS group, 32 cases (43.8%) underwent SLND, and the others received systematic PLND, in which 6 patients (8.2%) only had comprehensive lymphadenectomy on one side due to negative mapping or previous surgery. The LESS group only had 4 cases (7.8%) undergoing SLND, with the majority, 47 cases (92.2%), receiving systematic PLND.

**Table 1:** Baseline characteristics.

Variable	R-LESS (n=73)	LESS (n=51)	t/Z/χ2	p value
Age, mean (SD), years	47.8±10.9	45.3±9.1	-1.351	0.179
BMI, mean (SD), kg/m^2^	23.6±3.7	22.5±3.6	-1.570	0.119
Menopausal status, No. (%)				
No	42 (57.5)	35 (68.6)	1.570	0.210
Yes	31 (42.5)	16 (31.4)		
Gravidity, median (IQR)	3.0 (2.5)	4.0 (3.0)	-1.290	0.197
Parity, median (IQR)	1.0 (1.0)	2.0 (1.0)	-1.206	0.228
History of abdominal surgery, No. (%)				
No	36 (49.3)	27 (52.9)	0.158	0.691
Yes	37 (50.7)	24 (47.1)		
Comorbidity, No. (%)				
No	61 (83.6)	43 (84.3)	0.013	0.911
Yes	12 (16.4)	8 (15.7)		
Clinical stage (FIGO 2018), No. (%)				
IB1	46 (63.0)	35 (68.6)	1.474	0.479
IB2	20 (27.4)	14 (27.5)		
IIA1	7 (9.6)	2 (3.9)		

BMI, body mass index.

The R-LESS group experienced a significant reduction in both OT (236.5 ± 52.8 *vs* 390.8 ± 73.5 min, p<0.001) and EBL (median, 50.0 *vs* 150.0 mL, p<0.001) compared to the LESS group. Even for those who had systematic PLND bilaterally, R-LESS consistently enabled a substantial reduction in OT (259.3 ± 47.3 *vs* 397.0 ± 70.3 min, p<0.001). Regarding postoperative rehabilitation indexes, both groups showed comparable and favorable outcomes except for a shorter drainage time (3.7 ± 1.2 *vs* 4.7 ± 1.9 days, p=0.001) after R-LESS.

Four cases (7.8%) converted to multi-port laparoscopy in the LESS group due to difficult operation, whereas the R-LESS group achieved a complete success with all cases completed as planned without conversion. Three patients (4.1%) undergoing R-LESS encountered intraoperative complications including 1 bowel injury and 2 vascular injuries. In the LESS group, 5 patients (9.8%) experienced a total of 6 intraoperative complications including 2 bowel injuries and 1 bladder injury addressed with suturing repair, 2 ureteral stenoses requiring placement of ureteral stents under cystoscopy, and 1 vascular injury converting to multi-port laparoscopy because of excessive bleeding. The LESS group exhibited higher rates of intraoperative complication and conversion compared to the R-LESS group, though these results didn’t reach statistical significance (p>0.05).

Postoperative complication rates were also comparable (28.8% for R-LESS and 35.3% for LESS, p=0.441). Among those who underwent R-LESS, 1 patient required reoperation to evacuate an infected pelvic hematoma 30 days after surgery because of persistent fever, and 4 patients diagnosed with urogenital fistula by CT urography required ureteral reimplantation. In the LESS group, reoperations were needed for 4 patients, one of whom experienced intestinal perforation and 3 suffered from urogenital fistula (**Table 2**).

**Table 2:** Perioperative outcomes.

Variable	R-LESS (n=73)	LESS (n=51)	t/Z/χ2	p value
No-manipulation technique, No. (%)				
Zheng’s 4C suspension method	17 (23.3)	51 (100)	71.342	**<0.001**
Banding method	56 (76.7)	0 (0)		
Extent of lymphadenectomy, No. (%)				
SLND	32 (43.8)	4 (7.8)	26.464	<0.001^*^
Unilaterally systematic PLND	6 (8.2)	0 (0)		
Bilaterally systematic PLND	35 (47.9)	47 (92.2)		
Operative time, mean (SD), min	236.5±52.8	390.8±73.5	12.856	**<0.001**
Estimated blood loss, median (IQR), mL	50.0 (50.0)	150.0 (100.0)	-5.774	**<0.001**
Intraoperative complication, No. (%)	n=3 (4.1)	n=5 (9.8)	0.808	0.369
Bowel injury	1	2		
Bladder injury	0	1		
Ureteral injury	0	2		
Vascular injury	2	1		
Conversion to multi-port laparoscopy, No. (%)	0 (0)	4 (7.8)	3.671	0.055
Transfusion, No. (%)	1 (1.4)	0 (0)	NA	1.000^*^
Exhaust time, mean (SD), days	2.7±0.9	2.5±0.7	-1.266	0.208
Drainage time, mean (SD), days	3.7±1.2	4.7±1.9	3.512	**0.001**
Indwelling catheter time, median (IQR), days	21.0 (3.5)	21.0 (5.0)	-0.270	0.787
Postoperative length of stay, median (IQR), days	5.0 (1.5)	6.0 (1.0)	-1.706	0.088
Pain assessment with VAS scores, mean (SD)				
12 h	2.2±0.7	2.0±0.8	-1.080	0.282
24 h	2.0±0.6	1.9±0.7	-0.648	0.518
36 h	1.6±0.7	1.8±0.7	1.492	0.138
Postoperative complication, No. (%)	n=21 (28.8)	n=18 (35.3)	0.593	0.441
Infection (respiratory, pelvic, urinary infection and septicopyemia)	4	5		
Urinary retention	12	10		
Urogenital fistula	4	3		
Ileus	2	0		
Intestinal perforation	0	1		
Lymphocele infection/lymphedema	4	1		
Pulmonary embolism	1	1		

SLN, sentinel lymph node dissection; PLND, pelvic lymph node dissection.

^*^Calculated by Fisher exact test

No significant differences were observed between the two groups regarding histologic types, with squamous carcinoma (61.6% in R-LESS and 62.7% in LESS) being the predominant type. Besides, patients with poor differentiation (54.8% in R-LESS and 62.7% in LESS), deep stromal invasion (35.6% *vs* 31.4%), parametrial involvement (5.5% *vs* 2.0%), vaginal fornix involvement (17.8% *vs* 5.9%), positive margin (4.1% *vs* 0%), lymph node metastasis (9.6% *vs* 11.8%), and length of resected vagina after being fixed with 10% formalin (2.0 ± 0.6 *vs* 2.1 ± 0.5 cm) were also comparable (p≥0.05). However, the LESS group had significantly more patients with LVSI (49.0% *vs* 31.5%, p=0.049). For those who received bilaterally systematic PLND, there was no statistical difference in the mean number of retrieved nodes (28.8 ± 5.4 *vs* 30.4 ± 6.5, p=0.233). After integrating pathological information, the distributions of patients’ postoperative disease status were found to be similar between the two groups (p=0.544).

**Figure 2:**
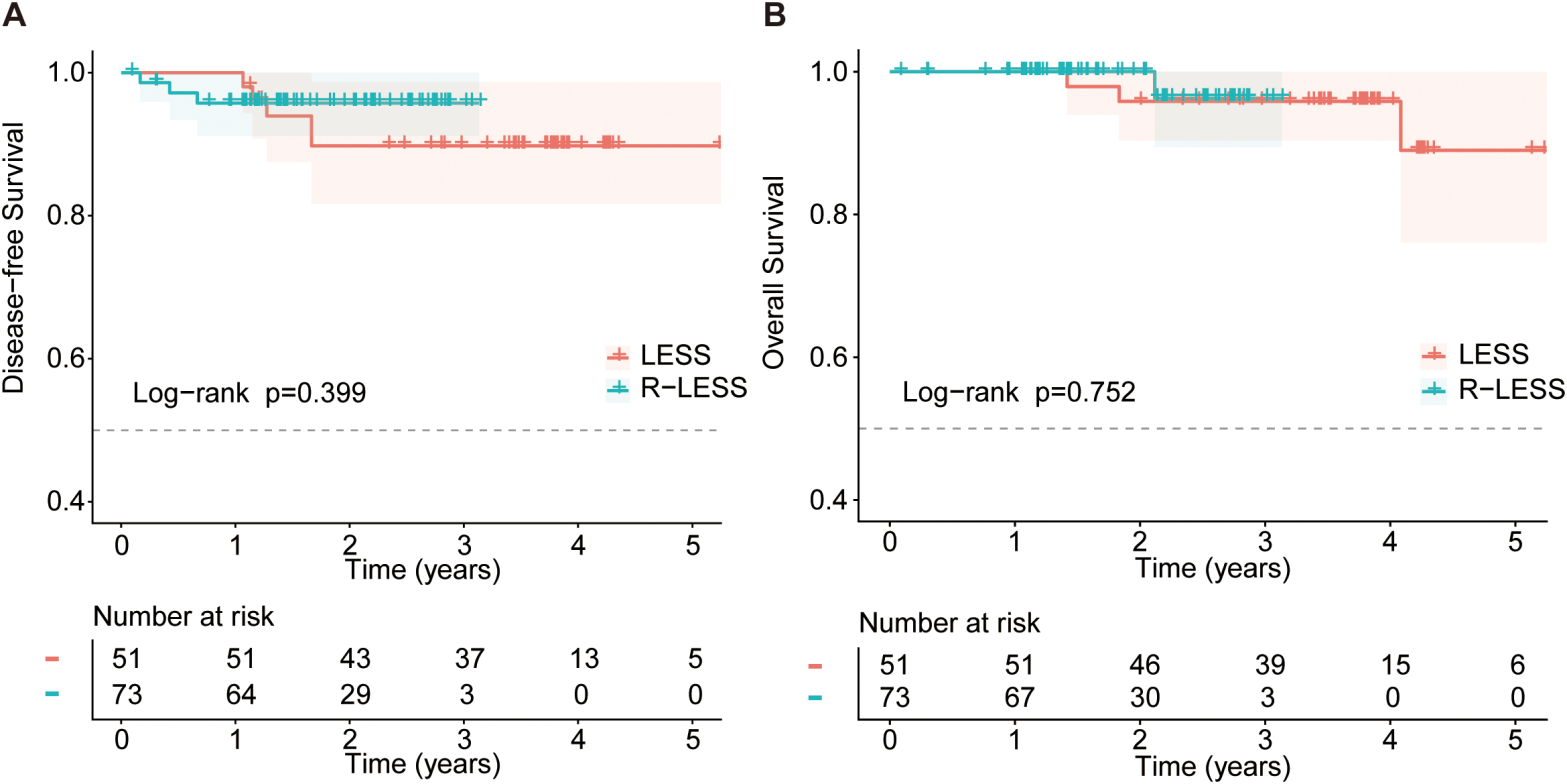
Comparisons of oncological outcomes between R-LESS and LESS **(A)** Disease-free survival; **(B)** Overall survival.

The determinations of adjuvant therapies were made by the surgeon and chemo-radiational oncologists as recommended by the National Comprehensive Cancer Network (NCCN) guidelines. No difference was found regarding the postoperative interventions (p=0.136). The median follow-up duration was 30.2 (95%CI, 25.4-35.0) months for all 124 enrolled patients, 18.9 (95%CI, 15.1-22.7) months for the R-LESS group, and 45.3 (95%CI, 44.4-46.2) months for the LESS group. Among the 5 patients with recurrence in the LESS group, sites included lung metastases (n=3), bone metastasis (n=1), and retroperitoneal lymph node metastasis (n=1). In the R-LESS group, the 3 recurrences consisted of vaginal cuff recurrence (n=1), lung metastasis (n=1), and bone metastasis (n=1). The recurrence rate (9.8% *vs* 4.1%) and death rate (5.9% *vs* 1.4%) after LESS were higher than those after R-LESS, but the differences didn’t reach statistical significance (p>0.05). The 4.5-year DFS of all 124 patients was 92.3%, and the 3-year OS was 96.3%. The 3-year DFS (95.8% for R-LESS *vs* 89.8% for LESS, p=0.399) and OS (96.3% *vs* 95.8%, p=0.752) were similar between the two groups (**Figure 2**, **Table 3**).

**Table 3:** Pathological information and survival outcomes.

Variable	R-LESS (n=73)	LESS (n=51)	t/χ2	p value
Histopathological type, No. (%)				
Squamous carcinoma	45 (61.6)	32 (62.7)	3.555	0.490^*^
Adenocarcinoma	16 (21.9)	12 (23.5)		
Adenosquamous carcinoma	8 (11.0)	4 (7.8)		
Neuroendocrine carcinoma	0 (0)	2 (3.9)		
Others	4 (5.5)	1 (2.0)		
Poor differentiation, No. (%)	40 (54.8)	32 (62.7)	0.779	0.377
Deep stromal invasion, No. (%)	26 (35.6)	16 (31.4)	0.241	0.623
Length of resected vagina, mean (SD), cm	2.0±0.6	2.1±0.5	1.117	0.266
Lymph-vascular space infiltration, No. (%)	22 (31.5)	25 (49.0)	3.881	**0.049**
Parametrial involvement, No. (%)	4 (5.5)	1 (2.0)	0.267	0.606
Vaginal fornix involvement, No. (%)	13 (17.8)	3 (5.9)	3.800	0.051
Positive margin, No. (%)	3 (4.1)	0 (0)	0.760	0.383
Number of resected nodes in bilateral systematic PLND, mean (SD)	28.8±5.4	30.4±6.5	1.202	0.233
Lymph node metastasis, No. (%)	7 (9.6)	6 (11.8)	0.151	0.697
Postoperative disease status, No. (%)				
IB1	38 (52.1)	33 (64.7)	3.167	0.544^*^
IB2	16 (21.9)	8 (15.7)		
IIA1	9 (12.3)	3 (5.9)		
IIB	3 (4.1)	1 (2.0)		
IIIC1p	7 (9.6)	6 (11.8)		
Adjuvant therapy, No. (%)				
None	41 (56.2)	32 (62.7)	4.856	0.136^*^
Radiotherapy	8 (11.0)	1 (2.0)		
Chemotherapy	0 (0)	1 (2.0)		
Concurrent chemoradiation	24 (32.9)	17 (33.3)		
Follow-up time, median (95% CI), months	18.9 (95%CI, 15.1-22.7)	45.3 (95%CI, 44.4-46.2)	91.602	**<0.001**
Recurrence, No. (%)	3 (4.1)	5 (9.8)	0.808	0.369
Death, No. (%)	1 (1.4)	3 (5.9)	0.780	0.377
3-year DFS	95.8%	89.8%	0.711	0.399
3-year OS	96.3%	95.8%	0.100	0.752

DFS, disease-free survival; OS, overall survival.

^*^Calculated by Fisher exact test

Cox proportional hazards regression was performed to analyze factors influencing disease recurrence and survival. On univariate analysis for DFS, histologic type, positive resection margin, lymph node metastasis, and postoperative pathological status were associated with an increased risk of recurrence (p<0.05). In the multivariate model, histologic type (HR 2.0, 95% CI 1.3-3.2; p=0.002) and lymph node metastasis (HR 16.8, 95% CI 3.7-75.1; p<0.001) remained independent risk factors for recurrence (**Table 4**). The wide confidence interval for lymph node metastasis may due to the small event number, and this result should be interpreted with caution. For OS, multivariate analysis identified histologic type (HR 2.5, 95% CI 1.3-4.9; p=0.005) as the only independent risk factor for death in this cohort (**Table 5**). The surgical approach (R-LESS *vs*. LESS) and no-manipulation technique adopted were not independent risk factors for either DFS or OS in the multivariate models.

**Table 4:** Univariate and multivariate Cox regression analysis for disease recurrence.

Variable	Univariate		Multivariate	
	HR (95%CI)	p value	HR (95%CI)	p value
Surgical approach	0.5 (0.1-2.3)	0.407		
No-manipulation technique	1.6 (0.4-6.6)	0.545		
Extent of lymphadenectomy	1.5 (0.4-6.1)	0.563		
Histopathological type	1.8 (1.2-2.8)	**0.009**	2.0 (1.3-3.2)	**0.002**
Differentiation	0.7 (0.2-2.9)	0.643		
Stromal invasion	2.3 (0.6-9.1)	0.245		
Lymph-vascular space infiltration	2.8 (0.7-11.8)	0.155		
Parametrial involvement	<0.1 (wide)	0.717		
Vaginal fornix involvement	2.5 (0.5-12.6)	0.253		
Positive margin	10.9 (1.3-89.1)	**0.026**		0.228
Lymph node metastasis	10.9 (2.7-44.1)	**<0.001**	16.8 (3.7-75.1)	**<0.001**
Postoperative disease status	1.9 (1.3-3.0)	**0.002**		0.931
Adjuvant therapy	1.4 (0.9-2.3)	0.156		

The bold values indicate statistical significance with p<0.05.

**Table 5:** Univariate and multivariate Cox regression analysis for disease-related death.

Variable	Univariate		Multivariate	
	HR (95%CI)	p value	HR (95%CI)	p value
Surgical approach	0.7 (0.1-7.6)	0.753		
No-manipulation technique	44.0 (wide)	0.447		
Extent of lymphadenectomy	12.9 (1.3-123.0)	**0.027**		0.202
Histopathological type	2.5 (1.3-4.9)	**0.005**	2.5 (1.3-4.9)	**0.005**
Differentiation	0.2 (<0.1-2.1)	0.194		
Stromal invasion	1.0 (0.1-10.1)	0.984		
Lymph-vascular space infiltration	1.7 (0.2-11.9)	0.606		
Parametrial involvement	17.2 (1.6-190.1)	**0.020**		0.326
Vaginal fornix involvement	4.1 (0.4-45.9)	0.249		
Positive margin	31.4 (2.8-351.8)	**0.005**		0.265
Lymph node metastasis	3.2 (0.3-31.1)	0.322		
Postoperative disease status	1.7 (1.0-2.9)	0.076		
Adjuvant therapy	2.1 (0.9-4.6)	0.069		

The bold values indicate statistical significance with p<0.05.

Given the observed significant difference in OT between the groups, an effect size (Cohen’s d) of 2.484 was calculated. With the actual sample sizes and a two-tailed α level of 0.05, the analysis yielded a statistical power exceeding 90%, indicating that the study was adequately powered for this important perioperative outcome.

## 4 Discussion

We presented our experience in two innovative no-manipulation techniques specifically utilized in transumbilical single-site radical hysterectomy for early-stage cervical cancer, which were designed to improve patients’ survival outcomes while preserving the benefits of MIS. They not only facilitated uterine manipulation but also achieved sufficient exposure of parametrial and LND areas.

In our study, the R-LESS group had significantly less OT and EBL, which was consistent with previous literatures. Gao et al. reported a comparative study of R-LESS and LESS in treating stage IB1 cervical cancer that showed remarkable advantages of R-LESS in shortening OT (223.56 ± 15.43 *vs* 248.61 ± 20.89 min, p<0.01) and reducing intraoperative bleeding (217.25 ± 16.77 *vs* 294.74 ± 24.00 mL, p<0.01) (11). Nie et al. reached the same conclusion in the comparison of robotic and laparoscopic multi-port surgery for radical hysterectomy (16). The high-definition 3D vision of da Vinci Xi system facilitated in clearly identifying and distinguishing parametrial structures, ureters and vessels, which cooperated with the flexible and stable instruments to perform dissection and resection quickly and precisely. The unipolar scissors, with the ability to provide sustaining energy stimulation, enhanced the efficiency of LND, offering a distinct advantage over the ultrasonic scalpel typically used in LESS. Moreover, the banding method was simpler and significantly reduced operative time with no need to adjust and replace the suspension sutures. Under the magnified view, any bleeding spot could be promptly identified and addressed with bipolar forceps, thus minimizing the time spent on frequent instrument exchanges that were often required in LESS. Furthermore, surgeons didn’t have to stand beside the operating table for hours of high-intensity work during R-LESS procedures, conserving both energy and physical strength that contributed to efficient and high-quality completion of the surgery.

Our findings indicated that patients experienced a quick recovery after single-site surgery, despite the LESS group had a longer duration of drainage due to a larger proportion of systematic PLND. In a previous study regarding radical hysterectomy for cervical cancer, no significant differences were observed between the robotic and laparoscopic groups in terms of catheter time, drainage time, exhaust time, and hospital stay (17). But the results from Luo et al. supported that the robotic surgery would promote a quicker recovery when compared to traditional laparoscopy (18). The placement of a pelvic drainage tube served a dual purpose of dynamically monitoring potential complications such as internal bleeding, lymphatic leakage, and ureteral fistula, as well as minimizing the risk of infection caused by pelvic effusions. The single-site surgery encouraged patients’ mobility by diminishing postoperative pain, which in return, facilitated drainage and bowel function recovery. Most patients were discharged the day after removing drainage tubes.

The intraoperative complication rate of the LESS group was slightly higher than that observed in the R-LESS group. Urinary injuries only occurred after LESS, and the incidence rate was in accordance with previous reports regarding MIS (19, 20). We speculated that the infiltration of cervical lesion and prior abdominal surgery caused dense adhesions of paravesical fossae and ureters to surrounding tissues, making it hard to distinguish anatomic structures under the two-dimensional vision provided by traditional laparoscopy. Moreover, the technical limitations inherent to LESS made it exceptionally difficult to perform meticulous dissection. Four conversions to multi-port laparoscopy all occurred in traditional LESS before the first case of R-LESS. Except for reduced surgical difficulty brought by the robotic system, the surgeon had accumulated considerable expertise in single-site radical hysterectomy prior to the initiation of R-LESS.

The postoperative complication rate was 28.8% for R-LESS and 35.3% for C-LESS with no statistical difference that were similar to Gallotta’s report regarding robotic and laparoscopic multi-port surgery (21). But there were also studies supported that the robotic surgery could significantly reduce postoperative complications following radical hysterectomy (16, 22). Urogenital fistula was the most severe complication after radical hysterectomy, potentially resulting from direct damage, delayed thermal injury, or accidental suture of the bladder or ureters during vaginal cuff closure. Postoperative radiotherapy could also lead to tissue ischemia, necrosis and fistula formation. The incidence rate of urogenital fistula in our study was similar to that reported in laparotomy (23), suggesting that the LESS approach would not increase the risk.

Numbers of retrieved nodes in both groups were more than 21.0-27.4 reported in laparotomic PLND (23, 24). Gao et al. had reached the same conclusion as ours that LESS had satisfying lymph node retrieval no matter whether it was assisted by the robotic system or not (21.37 ± 4.11 *vs* 20.71 ± 3.47, p=0.475) (11). However, another study indicated that robotic surgery had significantly more resected nodes in comparison to traditional multi-port laparoscopy (29.45 ± 9.78 *vs* 21.05 ± 11.22, p<0.001) (25). The enhanced vision and flexible instruments of robotic system helped inexperienced surgeons rapidly acquire proficiency in complex procedures, ensure the thoroughness of LND, and reduce missed diagnosis and recurrence.

The 4.5-year DFS and 3-year OS rates observed in all 124 patients were better than those reported for the laparoscopic group in LACC trial. Besides, previous studies have also provided evidence supporting that the survival outcomes and patterns of recurrence following robotic radical hysterectomy were not different from those of the laparotomic approach (26, 27). Given a relatively shorter follow-up time for the R-LESS group, a comparison was made only for the 3-year DFS and OS rates, which revealed no statistical significance. However, determining whether no-manipulation techniques could alter the controversies regarding MIS for cervical cancer will necessitate a larger cohort with extended follow-up data.

To our knowledge, this represents the first comparative study between R-LESS and LESS radical hysterectomy for early-stage cervical cancer employing two distinct no-manipulation techniques, both of which are easily implemented while ensuring adequate surgical excision. All procedures were performed by the same surgeon, thereby minimizing potential bias related to surgical skill and experience. This study also has several limitations. This study has several limitations. As a single−center retrospective analysis, the generalizability of our findings is inherently constrained by possible selection bias and institution−specific practices. Therefore, extrapolation of these results to broader populations should be made with caution. Although the sample size is substantial for a single−center series, it may still be underpowered to detect small but clinically meaningful differences, particularly in survival outcomes and complication rates. Furthermore, a temporal disparity in surgical dates between the two cohorts led to a significant difference in median follow−up duration, which could introduce bias into survival analyses. Continued follow-up is essential to confirm the durability of these promising early survival outcomes. Cohort studies comparing laparotomy and MIS with no-manipulation technique for radical hysterectomy with balanced follow-ups are expected to further validate its efficacy.

It should be noted that the widespread application of R-LESS must be contextualized within its economic implications and associated learning curve. The substantial capital investment required for the robotic system, alongside ongoing maintenance and instrument costs, presents a significant barrier to its broad accessibility. Patients’ financial capacity and acceptance of emerging technologies should be carefully considered. For those with economic hardship, traditional LESS using Zheng’s 4C suspension method remains a safe and viable alternative. Beyond yielding superior perioperative outcomes, another major advantage of R-LESS lies in its ability to enhance anatomical visualization while improving operational flexibility and precision. It enables assistance without requiring additional ports, thereby reducing the complexity of such complex procedures and shortening the learning curve.

This study demonstrated that R-LESS and LESS are both feasible and safe in radical hysterectomy with no-manipulation technique. R-LESS is superior to LESS with significantly reduced OT and EBL with a tendency of less conversions and complications. Both approaches show comparable short-to-medium survival outcomes when sufficient excision extents are reached assisted by valid no-manipulation techniques. However, definitive conclusions regarding oncologic non-inferiority require future investigation with larger sample sizes and longer follow-up. Moreover, prospective randomized studies comparing LESS no-manipulation technique with laparotomy are needed to further elucidate its safety and advantages.

## Data Availability

The original contributions presented in the study are included in the article/supplementary material. Further inquiries can be directed to the corresponding author.

## References

[1] Bray F Laversanne M Sung H Ferlay J Siegel RL Soerjomataram I . Global cancer statistics 2022: GLOBOCAN estimates of incidence and mortality worldwide for 36 cancers in 185 countries. CA: Cancer J Clin. (2024) 74:229–63. doi: 10.3322/caac.21834, PMID: 38572751

[2] Bhatla N Aoki D Sharma DN Sankaranarayanan R . Cancer of the cervix uteri: 2021 update. Int J Gynaecology Obstetrics. (2021) 155 Suppl 1:28–44. doi: 10.1002/ijgo.13865, PMID: 34669203 PMC9298213

[3] Ramirez PT Frumovitz M Pareja R Lopez A Vieira M Ribeiro R . Minimally invasive versus abdominal radical hysterectomy for cervical cancer. New Engl J Med. (2018) 379:1895–904. doi: 10.1056/NEJMoa1806395, PMID: 30380365

[4] Li P Chen L Ni Y Liu J Li D Guo J . Comparison between laparoscopic and abdominal radical hysterectomy for stage IB1 and tumor size <2 cm cervical cancer with visible or invisible tumors: A multicentre retrospective study. J Gynecologic Oncol. (2021) 32:e17. doi: 10.3802/jgo.2021.32.e17, PMID: 33470062 PMC7930457

[5] Chen C Wang L Fan H Lin B Xu Y Ma B . Long-term oncological outcomes of laparoscopic versus abdominal surgery in stage A1(LVSI+) - B1 cervical cancer patients -- a real-world study. Chin J Prac Gynecol Obste. (2020) 36:637–64. doi: 10.19538/j.fk2020070115

[6] Kohler C Hertel H Herrmann J Marnitz S Mallmann P Favero G . Laparoscopic radical hysterectomy with transvaginal closure of vaginal cuff - a multicenter analysis. Int J Gynecological Cance. (2019) 29:845–50. doi: 10.1136/ijgc-2019-000388, PMID: 31155516

[7] Su MC Zheng Y Yang F Liu YY . Placement of robotic single-site surgery with the tumor-free technique for early cervical cancer using the da vinci xi platform. Asian J Surg. (2023) 46:1492–3. doi: 10.1016/j.asjsur.2022.09.049, PMID: 36184286

[8] Peng S Zheng Y Yang F Wang K . Transumbilical single-port laparoscopic radical hysterectomy with pelvic lymphadenectomy without uterine manipulator: report of 37 cases. Chin J Minim Invasive Surg. (2022) 22:722–7. doi: 10.3969/j.issn.1009-6604.2022.09.007

[9] Boruta DM . Laparoendoscopic single-site surgery in gynecologic oncology: An update. Gynecologic Oncol. (2016) 141:616–23. doi: 10.1016/j.ygyno.2016.03.014, PMID: 26980644

[10] Chen S Qi X Chen L Li F Wang N Wang Y . Laparoendoscopic single-site radical hysterectomy: Sufficient exposure via effective suspension. J Minimally Invasive Gynecology. (2020) 27:809–10. doi: 10.1016/j.jmig.2019.08.030, PMID: 31518713

[11] Gao J Dang J Chu J Liu X Wang J You J . A comparative analysis of robotic single-site surgery and laparoendoscopic single-site surgery as therapeutic options for stage IB1 cervical squamous carcinoma. Cancer Manage Res. (2021) 13:3485–92. doi: 10.2147/CMAR.S299827, PMID: 33911898 PMC8071700

[12] Jang TK Chung H Kwon SH Shin SJ Cho CH . Robotic single-site versus multiport radical hysterectomy in early stage cervical cancer: An analysis of 62 cases from a single institution. Int J Med robotics Comput assisted Surg. (2021) 17:e2255. doi: 10.1002/rcs.2255, PMID: 33817949 PMC8365681

[13] Song C Jang TK Kong S Kang H Kwon SH Cho CH . Robotic single-site radical hysterectomy for early cervical cancer: A single center experience of 5 years. J Personalized Med. (2023) 13:733. doi: 10.3390/jpm13050733, PMID: 37240903 PMC10221641

[14] Wang K Chen S Zheng Y . Application of Zheng’s 4C suspension method in the transumbilical single-site surgery (TU-LSSS) for gynecologic oncology. Chin J Laparo Surg. (2021) 14:5–9. doi: 10.3877/cma.j.issn.1674-6899.2021.01.002

[15] Chen Y Zheng Y Xu LF Chen L . Zheng’s anchor suturing technique for safe and cosmetic umbilical incision in transumbilical laparoendoscopic single-site surgeries. Surg Today. (2023) 53:274–7. doi: 10.1007/s00595-022-02585-6, PMID: 36242640 PMC9876851

[16] Nie JC Yan AQ Liu XS . Robotic-assisted radical hysterectomy results in better surgical outcomes compared with the traditional laparoscopic radical hysterectomy for the treatment of cervical cancer. Int J Gynecological Cancer. (2017) 27:1990–9. doi: 10.1097/IGC.0000000000001101, PMID: 28858908 PMC5671798

[17] Huang N Xiao L Ma D Yu R . Comparison between robot-assisted and conventional laparoscopic radical hysterectomy. Chin J Robot Surg. (2020) 1:77–85. doi: 10.12180/j.issn.2096-7721.2020.02.001

[18] Luo C Liu M Li X . Efficacy and safety outcomes of robotic radical hysterectomy in Chinese older women with cervical cancer compared with laparoscopic radical hysterectomy. BMC women’s Health. (2018) 18:61. doi: 10.1186/s12905-018-0544-x, PMID: 29716555 PMC5930733

[19] Wright JD Herzog TJ Neugut AI Burke WM Lu YS Lewin SN . Comparative effectiveness of minimally invasive and abdominal radical hysterectomy for cervical cancer. Gynecologic Oncol. (2012) 127. doi: 10.1016/j.ygyno.2012.06.031, PMID: 22735788

[20] Uppal S Rebecca Liu J Kevin Reynolds R Rice LW Spencer RJ . Trends and comparative effectiveness of inpatient radical hysterectomy for cervical cancer in the United States, (2012-2015). Gynecologic Oncol. (2019) 152:133–8. doi: 10.1016/j.ygyno.2018.09.027, PMID: 30424895

[21] Gallotta V Conte C Federico A Vizzielli G Gueli Alletti S Tortorella L . Robotic versus laparoscopic radical hysterectomy in early cervical cancer: A case matched control study. Eur J Surg Oncol. (2018) 44:754–9. doi: 10.1016/j.ejso.2018.01.092, PMID: 29422253

[22] Chen L Liu LP Wen N Qiao X Meng YG . Comparative analysis of robotic vs laparoscopic radical hysterectomy for cervical cancer. World J Clin cases. (2019) 7:3185–93. doi: 10.12998/wjcc.v7.i20.3185, PMID: 31667168 PMC6819296

[23] Ceccaroni M Roviglione G Malzoni M Cosentino F Spagnolo E Clarizia R . Total laparoscopic vs. conventional open abdominal nerve-sparing radical hysterectomy: Clinical, surgical, oncological and functional outcomes in 301 patients with cervical cancer. J Gynecologic Oncol. (2021) 32:e10. doi: 10.3802/jgo.2021.32.e10, PMID: 33300311 PMC7767655

[24] Bogani G Cromi A Uccella S Serati M Casarin J Pinelli C . Laparoscopic versus open abdominal management of cervical cancer: Long-term results from a propensity-matched analysis. J Minimally Invasive Gynecology. (2014) 21:857–62. doi: 10.1016/j.jmig.2014.03.018, PMID: 24699300

[25] Han L Yan P Yao L Liu R Shao R Liu J . Safety and effectiveness of robotic hysterectomy versus conventional laparoscopic hysterectomy in patients with cervical cancer in China. Arch gynecology obstetrics. (2019) 300. doi: 10.1007/s00404-019-05148-2, PMID: 31006844

[26] Corrado G Cutillo G Saltari M Mancini E Sindico S Vici P . Surgical and oncological outcome of robotic surgery compared with laparoscopic and abdominal surgery in the management of locally advanced cervical cancer after neoadjuvant chemotherapy. Int J Gynecological Cancer. (2016) 26:539–46. doi: 10.1097/IGC.0000000000000646, PMID: 26825826

[27] Shah CA Beck T Liao JB Giannakopoulos NV Veljovich D Paley P . Surgical and oncologic outcomes after robotic radical hysterectomy as compared to open radical hysterectomy in the treatment of early cervical cancer. J Gynecologic Oncol. (2017) 28:e82. doi: 10.3802/jgo.2017.28.e82, PMID: 29027400 PMC5641532

